# Theory of Mind and Context Processing in Schizophrenia: The Role of Social Knowledge

**DOI:** 10.3389/fpsyt.2015.00098

**Published:** 2015-07-03

**Authors:** Maud Champagne-Lavau, Anick Charest

**Affiliations:** ^1^Aix-Marseille Université, CNRS, LPL UMR7309, Aix-en-Provence, France; ^2^Department of Psychiatry, University of Montreal, QC, Canada; ^3^Hôpital du Sacré-Cœur de Montréal, Pavillon Albert-Prévost, Montreal, QC, Canada

**Keywords:** theory of mind, ironic intent, social knowledge, context, stereotypes, social perception, schizophrenia

## Abstract

The present study sought to determine whether social knowledge such as speaker occupation stereotypes may impact theory of mind (ToM) ability in patients with schizophrenia (SZ). Thirty individuals with SZ and 30 matched healthy control (HC) participants were tested individually on their ToM ability using a paradigm showing that stereotypes such as speaker occupation influences the extent to which speaker ironic intent is understood. ToM ability was assessed with open questions on the speaker ironic intent, irony rating, and mockery rating. Social perception was also assessed through politeness rating. The main results showed that SZ participants, like HC participants, were sensitive to the social stereotypes. They used these stereotypes adequately to attribute mental states such as speaker ironic intent to a protagonist while they found it difficult to explicitly judge and attribute negative attitude and emotion, as evidenced by mockery rating. No difference was found between the two groups regarding social perception ability. These performances were not associated with clinical symptoms. The integration of contextual information seems to be a good target for cognitive remediation aiming to increase social cognition ability.

## Introduction

A deficit in social cognition – including theory of mind (ToM), emotion processing, social perception, and attributional bias – is one of the most disabling clinical characteristics of schizophrenia (SZ) (1). For this reason, social cognition has become a high priority domain of research for the study of SZ (2). It has been included as one of the seven domains represented in the MATRICS Consensus Cognitive Battery for clinical trials in SZ (2). In addition, results from recent meta-analysis and literature review have pointed out that there are strong correlations between social cognition and outcome, underlying that these correlations were strongest with ToM and, to a lesser degree, with social perception (1, 3). Thus, given the prominence of the impairments in social functioning in SZ, it is important to understand the cognitive processes underlying impaired social cognition in this disorder. This paper focuses on two aspects of social cognition, namely ToM and social perception, and particularly on the impact of social contextual information on ToM abilities in SZ.

Theory of mind is the ability to form representations of other people’s mental states (e.g., intention, thought, belief) and to use these representations to understand, predict, and judge their statements and behaviors (4, 5). A number of studies have reported impaired ToM ability in patients with SZ [see Ref. (6–9) for a review] assessed in natural communication situations (10, 11) and also in tasks assessing first- and second-order false beliefs (12–15), irony comprehension (16–21), hinting comprehension (22–24), and picture-sequencing tasks (25, 26). This ToM impairment in SZ patients has been supposed to result from their inability to process contextual information (27–31). But research investigating this hypothesis in SZ has only used non-social contextual information using different kinds of visual and verbal tasks (27, 32). In line with the findings of Uhlhaas et al. (32) and Green et al. (29, 30), Champagne-Lavau et al. (21) showed that participants with SZ correctly perceived contextual information, but they showed difficulty in correctly integrating this information, performing significantly worse than healthy participants when attributing mental states.

Few studies (33) have dealt with the role of social knowledge in ToM ability. However, social knowledge is also a kind of contextual information that contributes to the attribution of mental states to others (34). Social knowledge refers “to awareness of the roles, rules, and goals that characterize social situations and guide social interactions” [(35): p. 1212, Ref. (1, 33, 36)].

To our knowledge, only the work by Corrigan and Penn and their colleagues (see below) directly investigated the processing of social knowledge and social perception in SZ. Social perception refers to the ability to identify social cues from the behavior of others in a given social context. Tasks used to investigate social perception abilities generally assess the ability of a person to identify social roles, social context, and rules of a society (37, 38). They require to process social cues which can be concrete (i.e., actions, roles, and dialogs explicitly displayed by the protagonists) or abstract (i.e., knowledge inferred from action and dialog of the protagonists such as affects, rules, and goals) [(39, 40), see Ref. (41) for examples]. The authors found that, compared to healthy participants, individuals with SZ were impaired in social perception (42). These disorders have been related to a difficulty in processing the social context (38). Thus, because of their difficulties in identifying social information, individuals with SZ would have difficulties in recognizing the rules and social conventions associated with a given situation (41, 43). These difficulties would rather concern the abstract social cues than the concrete ones (39, 43). Moreover, these difficulties would be relatively independent of the symptomatology (38).

The present study investigated whether social knowledge, which is a kind of social contextual information, impacts the attribution of mental states to others. Irony understanding is a relevant paradigm to assess this issue in SZ, since social knowledge such as speaker occupation stereotypes is contextual information that has been found to influence the attribution of speaker intent (ToM) in healthy individuals (44). Stereotypes are knowledge people have on other people as members of a group (34, 45). They are stored in memory, processed and activated automatically and unconsciously (45, 46). They were shown to be present even in people with limited social experience and in people with impaired social behavior such as children with autism (47).

Regarding irony processing, it has been demonstrated that, among several factors (e.g., level of incongruity between context and speaker’s utterance, prosody), speaker features (e.g., his/her social status or his/her occupation) influence the extent to which ironic intent is perceived among healthy subjects (44, 48–50). More precisely, Katz and Pexman (50) pointed out that people perceive members of certain occupations as likely to use irony while members of other occupations are perceived as unlikely to use irony. For example, one is more likely to interpret a statement as ironic and mocking if it is said by an actor rather than a clergyman (44, 50). According to Pexman and Olineck (44), “the occupation stereotype contributes to the ironic environment by indicating that the speaker is likely to have a negative attitude (tendency to be critical) and that such an attitude is likely to be indirectly expressed (through humor and insincerity)” (page 268). These authors also reported that saying something negative in an ironic way (i.e., “you are a wonderful singer” in a context where you sang and people started to throw things at you) is perceived as more polite than saying something in a direct way (i.e., “you are a horrible singer”). Thus, assessing the degree of perceived politeness is a way to assess social perception.

These findings related to social knowledge and social perception were used in the present study to determine whether social knowledge influences the attribution of intention by patients with SZ. As far as we know, no research investigating ToM ability via irony comprehension in SZ has assessed the impact of social knowledge on such ability. Accordingly, the present study aimed at determining whether social knowledge such as stereotypes (type of speaker’s occupation) – which has been demonstrated to be social factors that cue speaker ironic intent in healthy individuals – also cues comprehension of ironic intent in SZ. It also aimed at assessing social perception in SZ participants. To these aims, we used a task of irony understanding manipulating contextual information according to the presence of a speaker occupation cueing or not ironic intent. Based on previous results, we hypothesized that difficulty in using social contextual data that cue speaker ironic intent will have an impact on the ToM ability of SZ participants.

## Materials and Methods

### Participants

The sample consisted of 60 participants: 30 individuals with SZ and 30 healthy control (HC) participants with no history of psychiatric disorders. SZ patients were diagnosed by clinicians according to DSM-IV diagnostic criteria. All SZ participants were outpatients who had been recruited from the *pavillon Albert prévost of the Hôpital du Sacré-Coeur of Montréal*. They were stable and on antipsychotic medication with a normal recommended range of dosage (the average chlorpromazine equivalent was 642 ± 651 mg/day). The severity of symptoms was measured using the Positive and Negative Symptom Scale [PANSS; (51)]. HC participants had been recruited in the local community. They were matched with the SZ patients for age and educational level (cf. Table 1 for the demographic data). The two groups did not significantly differ with regard to age [*t*(58) = −0.94, *p* > 0.05], educational level [*t*(58) = −0.65, *p* > 0.05], and IQ estimated using the NART (52) [*t*(58) = −0.74, *p* > 0.05]. All participants were native French-speakers with no previous neurological history.

**Table 1:** **Demographic and clinical data**.

	Schizophrenia		Healthy control		*p*-value
	Mean	SD	Mean	SD	
Age	43.9	8.3	41.7	9.6	0.94
Educational level	12.8	1.9	13.2	2.4	0.80
Gender (male/female)	(17/13)		(10/20)		
Duration of illness	17.3	8.9			
PANSS (positive)	16.3	5.8			
PANSS (negative)	15.8	6.3			
PANSS (general)	33.4	10.2			
NART	37.7	7	35.9	5.5	0.54

Written consent forms were obtained from all participants, according to ethics guidelines set out by the University of Montreal and the *Hôpital du Sacré-Coeur de Montréal*.

### Measures

A task of irony understanding was used to test the attribution of intention. The stimuli were composed of 48 stories from Champagne-Lavau et al. (21) controlled for familiarity and plausibility. To assess how social knowledge, such as occupation stereotypes, influenced participants’ ToM ability, the context was manipulated according to the presence of an occupation cueing or not ironic intent (cf. Supplementary Material, for example). Following Pexman and Olineck (44), a no occupation condition was also devised to create filler stimuli. This condition, in which the speakers were identified by their first names, was included to prevent the participants from developing response strategy that would help them foresee job information. The speaker occupations were chosen following a pilot study conducted with the procedure used by Pexman and Olineck (44). Forty undergraduate students from the University of Montreal were recruited for this pilot study. For each occupation, they were asked to imagine that a member of that occupation got a flat tire on the way to work. They were then asked to rate, on a 7-point scale (1 = low probability, 7 = high probability), the likelihood that the member of this occupation would make an ironic remark about the situation. Forty five occupations were tested in that pilot study. The following eight occupations were judged as having the highest probability (*p* > 4.50) of ironic remarks (“sarcastic occupations”): *comedian*, *talk show host*, *actress*, *artist*, *movie critic, mechanic*, *plumber*, and *insurance agent* (*M* = 4.91, SD = 0.43). The following eight occupations were judged as having the lowest probability (*p* < 3.50) of ironic remarks (“non-sarcastic occupations”): *accountant*, *clergyman*, *scientist*, *librarian*, *soldier*, *waiter*, *bank teller*, and *veterinarian* (*M* = 2.78, SD = 0.30). These two occupation conditions were significantly different [*t*(14) = 11.49; *p* < 0.0001]. The stories ended either with a literal statement (e.g., *Marie has a poor memory*) or with an ironic statement (e.g., *Marie has a phenomenal memory*) to prevent judgments at chance level. Thus, the materials for the experiment were 48 stories involving a 2 statements (ironic or literal) by 3 occupations (occupation that cues ironic intent, occupation that does not cue ironic intent, and no occupation) combination of conditions (cf. Supplementary Material, for example). The stories were presented in random order. To control for prosody, stimuli were presented on a sheet of paper. Each stimulus was placed in front of the participant and remained there throughout the reading and questioning so that participants did not have to remember it. This was done in order to minimize memory and attention requirements.

In line with the study by Pexman and Olineck (44), participants were asked to read each of these 48 stimuli and then answer the following question: “What does X (the speaker) really mean?” Then, they had to judge on a 7-point scale if the speaker was being ironic (1 = not at all ironic, 7 = extremely ironic), if he/she was mocking someone (1 = not at all mocking, 7 = extremely mocking), and if he/she was saying something polite (1 = not at all polite, 7 = extremely polite). Rating scales on irony and mockery were used to assess attribution of speaker intent while the question on politeness was used to assess social perception.

All participants were tested individually over one session in a quiet room.

### Data analysis

The data were analyzed using 2 groups (SZ, HC) × 2 statements (ironic, literal) × 2 speaker occupations (sarcastic, non-sarcastic) repeated-measures ANOVA. A Spearman correlation analysis was conducted on the performances (i.e., percentage of correct responses to the question on the speaker iconic intent, irony rating scale, mocking rating scale, politeness rating scale) of the SZ group for each condition to examine the relationship between ToM and symptoms. The alpha level was set at *p* < 0.05 for all the analyses.

## Results

### Group comparison on theory of mind

#### Percentage of Correct Responses to the Open Question

**Figure 1:**
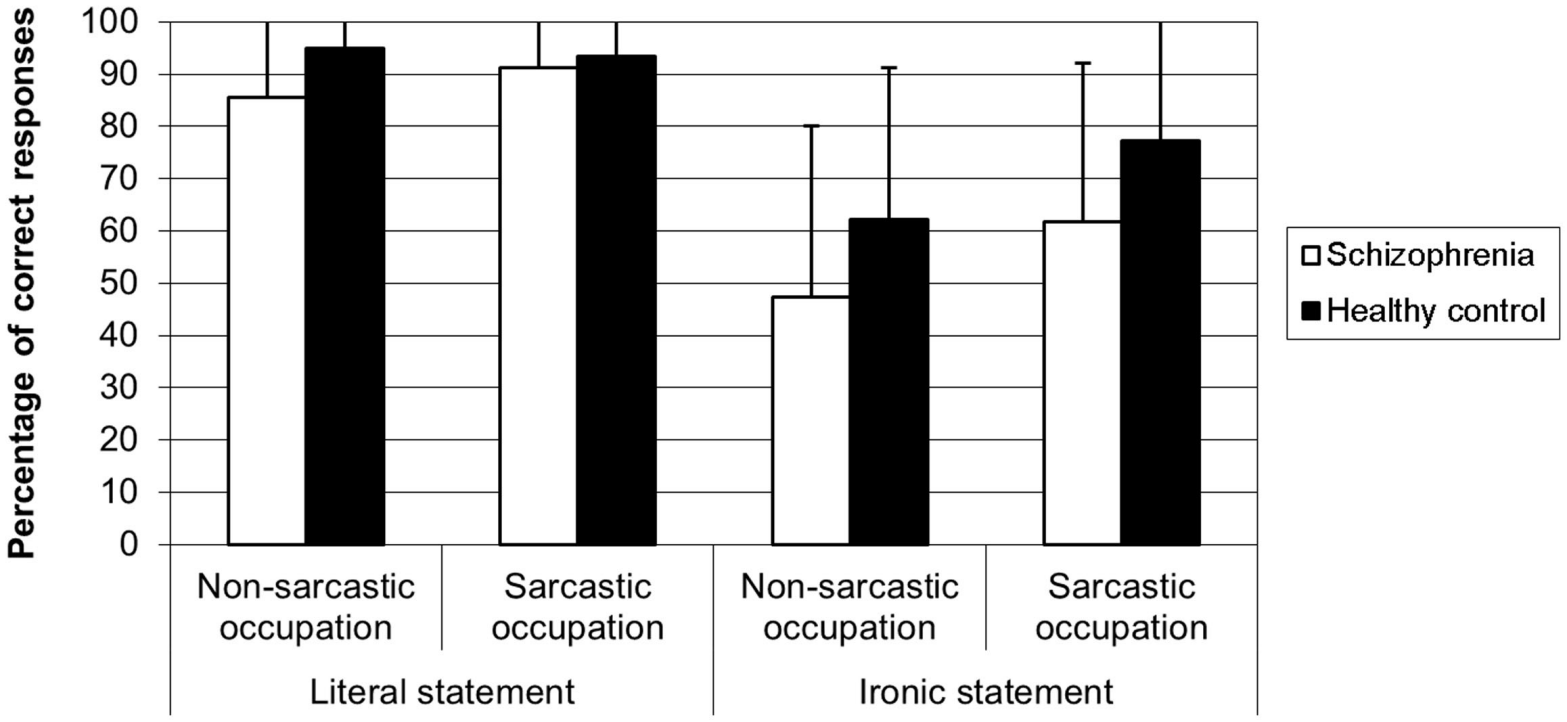
**Percentage of correct responses to the open question on ironic speaker intent in schizophrenia and healthy control participants**.

The 2 × 2 × 2 ANOVA on the percentage of correct responses to the questions on the speaker intent revealed a significant main effect of group [*F*(1, 58) = 6.52, *p* < 0.01], showing that SZ patients (71.9%) made more errors than HC participants (81.94%). There was a significant main effect of speaker occupation [*F*(1, 58) = 21.81, *p* < 0.0001]. The percentage of correct responses was higher when the speaker had a sarcastic occupation than when he/she had a non-sarcastic occupation. There was also a significant main effect of statement [*F*(1,58) = 58.58, *p* < 0.0001] showing a higher percentage of correct responses for the literal statements than for the ironic ones. The speaker occupation × statement interaction was significant [*F*(1,58) = 17.12, *p* < 0.001] revealing that, for ironic statements, irony ratings were higher when the speaker had a sarcastic occupation than when he/she had a non-sarcastic occupation (*p* < 0.0001) whatever the group, while for literal statements such difference did not exist (*p* > 0.05). The group × speaker occupation × statement [*F*(1,58) = 1.59, *p* > 0.05], the group × statement [*F*(1,58) = 1.54, *p* > 0.05], and the speaker occupation × statement interactions [*F*(1,58) = 0.87, *p* > 0.05] were not significant (cf. Figure 1).

#### Rating Scales

##### Irony rating

**Figure 2:**
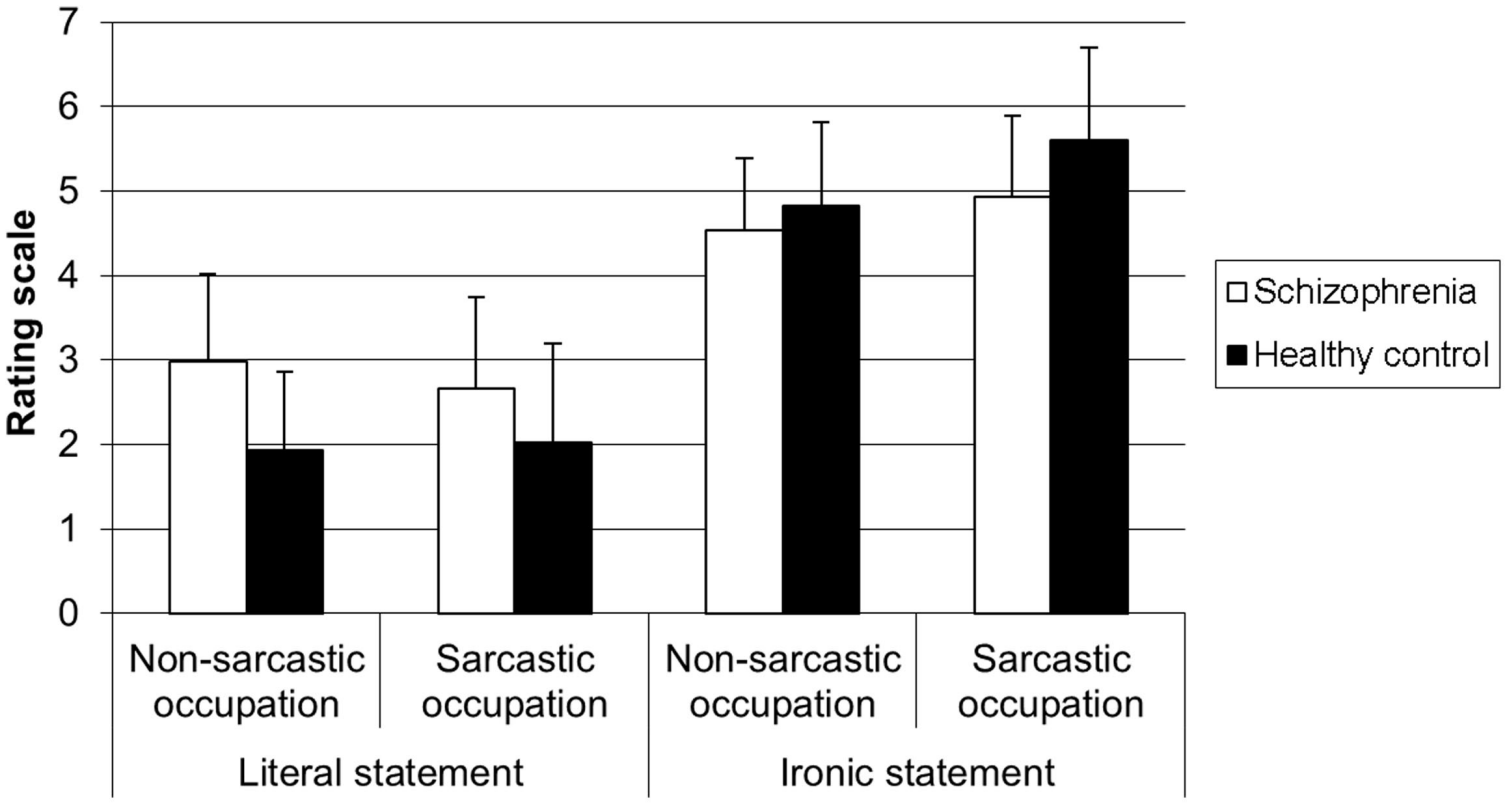
**Level of irony of statement for each type of occupation according to the type of participant**.

The 2 × 2 × 2 ANOVA on the ratings of the extent to which the speaker was ironic revealed a significant main effect of speaker occupation [*F*(1, 58) = 9.20, *p* < 0.01]. Irony ratings were higher when the speaker had a sarcastic occupation than when he/she had a non-sarcastic occupation. There was also a significant main effect of statement [*F*(1,58) = 196.46, *p* < 0.0001] showing that irony ratings were higher when the statement was ironic than when it was literal. There was no main effect of group [*F*(1, 58) = 1.52, *p* > 0.05]. The group × speaker occupation × statement interaction was not significant [*F*(1, 58) = 0.02, *p* > 0.05]. The speaker occupation × statement interaction was significant [*F*(1, 58) = 14.71, *p* < 0.0001], showing that, for ironic statements, irony ratings were higher when the speaker had a sarcastic occupation than when he/she had a non-sarcastic occupation (*p* < 0.0003) whatever the group, while for literal statements such difference did not exist (*p* > 0.05). The speaker occupation × group interaction was also significant [*F*(1,58) = 6.62, *p* < 0.01], revealing that, whatever the type of statement, there was no difference between the sarcastic and non-sarcastic occupation conditions (*p* > 0.05) in the SZ group, while such difference was present in the HC group (*p* < 0.001). And finally, the group × statement interaction was significant [*F*(1,58) = 12.91, *p* < 0.001]. In both groups, irony ratings were higher when the statement was ironic than when it was literal (SZ group: *p* < 0.001; HC group: *p* < 0.001) (cf. Figure 2).

##### Mocking rating

**Figure 3:**
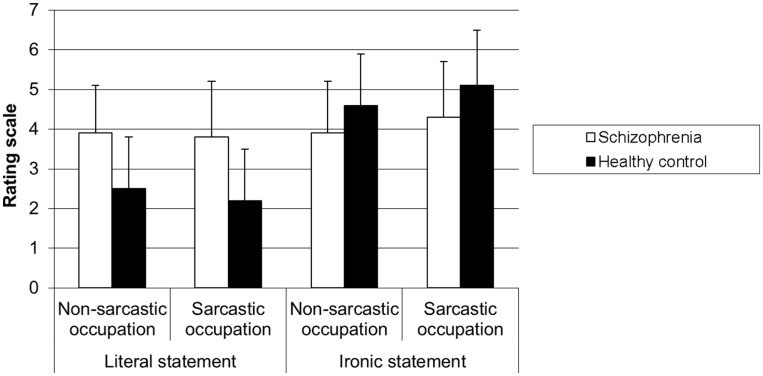
**Level of mockery of statement for each type of occupation according to the type of participant**.

The 2 × 2 × 2 ANOVA on the ratings of the extent to which the speaker was mocking someone revealed a main effect of the statement [*F*(1,58) = 30.70, *p* < 0.001] showing that mocking ratings were higher for the ironic statements than for the literal ones. There was a marginal effect of group [*F*(1,58) = 3.43, *p* = 0.06] and no main effect of speaker occupation [*F*(1,58) = 2.22, *p* > 0.05]. The speaker occupation × statement interaction was significant [respectively *F*(1, 58) = 12.10, *p* < 0.001] revealing that, for ironic statements, irony ratings were higher when the speaker had a sarcastic occupation than when he/she had a non-sarcastic occupation (*p* < 0.0001) whatever the group, while for literal statements such difference did not exist (*p* > 0.052). The group × statement interaction was significant [*F*(1,58) = 20.10, *p* < 0.0001]. In the SZ group, the mocking ratings were the same for the literal statements and the ironic ones (*p* > 0.05), while in the HC group, the mocking ratings were higher for the ironic statements than for the literal ones (*p* < 0.001), whatever the speaker occupation. The group × speaker occupation × statement interaction [*F*(1, 58) = 0.37, *p* > 0.05] and the group × speaker occupation interaction [*F*(1, 58) = 0.60, *p* > 0.05] were not significant (cf. Figure 3).

To sum up, patients with SZ had lower performances than HC participants on the open question on the speaker intent in all the conditions. This does not lead to conclude that they have any impairment in attributing ironic intent. Using the irony rating scales, they showed performances similar to those of HC control participants, except they rated the statements in the same way for both speaker occupation conditions, whatever the type of statement. Surprisingly, SZ patients also judged mockery in the same way in all the conditions, meaning they rated both ironic and literal statements as much as mocking (around 4), by contrast to HC participants who rated ironic statements as more mocking than the literal ones.

### Group comparison on social perception

#### Politeness Rating

**Figure 4:**
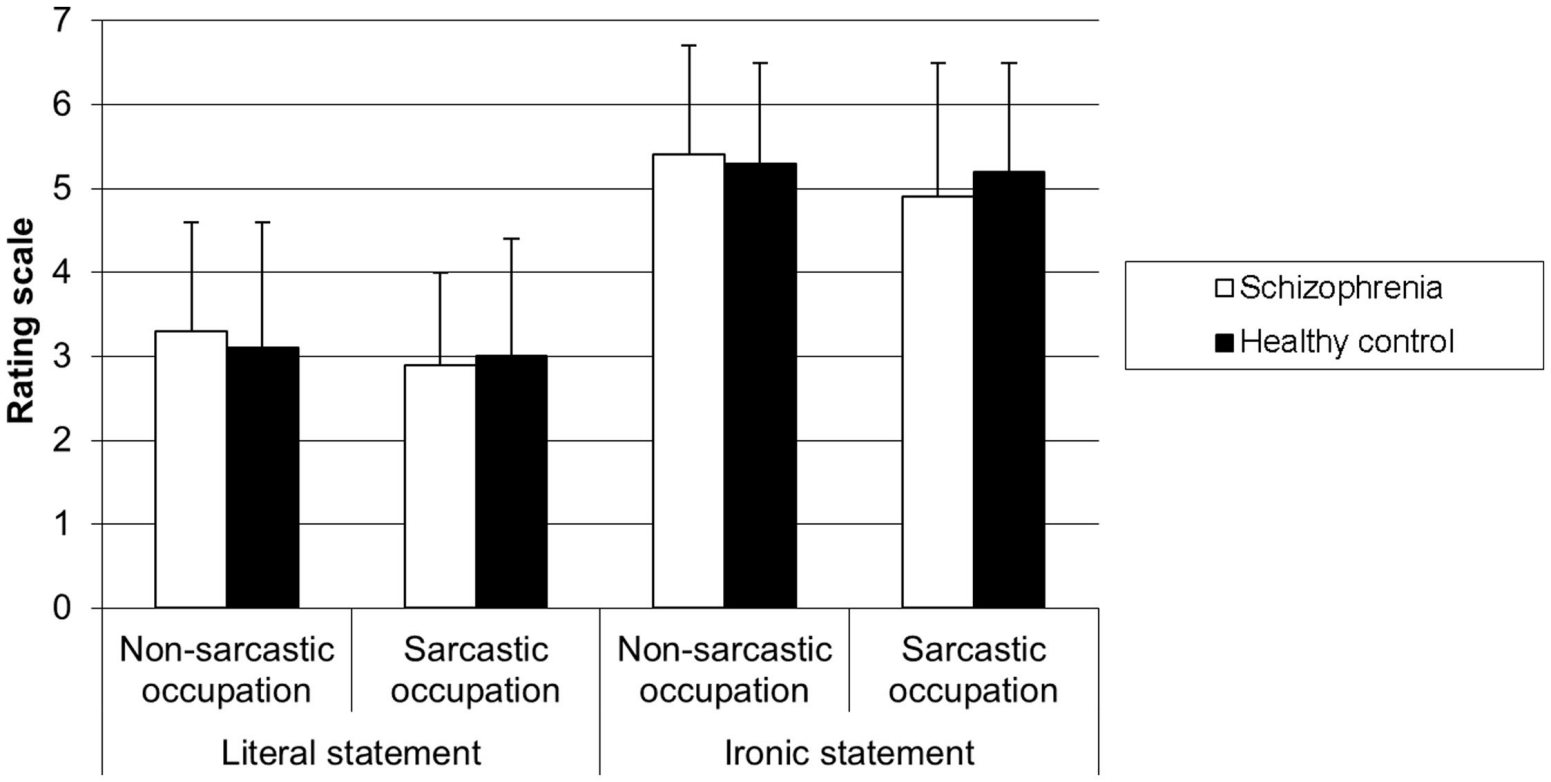
**Level of politeness of statement for each type of occupation according to the type of participant**.

The 2 × 2 × 2 ANOVA on the ratings of the extent to which a speaker said something polite revealed a significant main effect of speaker occupation [*F*(1, 58) = 19.54, *p* < 0.0001] showing that politeness ratings were higher when the speaker had a non-sarcastic occupation than when he/she had a sarcastic occupation. There was also a significant main effect of statement [*F*(1,58) = 98.66, *p* < 0.0001] showing that politeness ratings were higher when the statements were ironic than when they were literal. There was no main effect of group [*F*(1,58) = 0.03, *p* > 0.05]. The group × speaker occupation interaction was significant [*F*(1,58) = 5.77, *p* < 0.05], showing that in the SZ group, the politeness ratings were higher when the speaker had a non-sarcastic occupation than when he/she had a sarcastic occupation (*p* < 0.0002), while such difference did not exist in the HC group (*p* > 0.05), whatever the type of statement. The group × occupation × statement [*F*(1,58) = 0.16, *p* > 0.05], the group × statement [*F*(1,58) = 0.03, *p* > 0.05] and the occupation × statement interactions [*F*(1,58) = 0.32, *p* > 0.05] were not significant (cf. Figure 4).

##### Association of ToM performances with clinical symptoms

No correlation was found between ToM performances and PANSS in the SZ group.

## Discussion

The present study sought to determine whether social knowledge, such as speaker occupation stereotypes, may impact ToM ability in patients with SZ. We used a psycholinguistic paradigm showing that stereotypes, such as speaker occupation, influence the extent to which speaker ironic intent is understood. ToM ability was assessed with open questions on the speaker ironic intent, irony rating, and mockery rating while social perception was assessed with politeness rating.

The main results revealed that, like HC participants, SZ participants were sensitive to the social stereotypes when they judged the statements and when they answered the open question on the speaker ironic intent. Indeed both groups found that the speaker meant something ironic more often when the speaker had a sarcastic occupation than when he had a non-sarcastic occupation. Interestingly, SZ participants seemed to adequately use these stereotypes to cue ironic intent since they did not show specific difficulties in attributing ironic intent to the speaker of the stories. Indeed, although they performed worse than HC participants, committing more errors than HC participants in answering the open questions, there was no difference across the different conditions. While such result disagrees with that of previous studies showing ToM difficulty with paradigms involving irony or sarcasm understanding (17–21), it is consistent with the study of Varga et al. (53) and with the results of Rapp et al. (54) finding relatively good performances (85% of correct responses) in SZ.

Surprisingly, while we expected the same performances for irony rating and mockery rating, since both scales are supposed to measure attribution of mental states, the results showed that, in contrast to HC participants, SZ patients judged the extent to which the speaker was mocking in the same way whatever the four conditions. Finally, SZ participants did not show any difficulty regarding social perception according to politeness rating in the present study.

### Influence of the knowledge of social stereotypes on ToM ability

The main results of the present study concur with previous findings in healthy individuals, showing that ironic statements uttered by a speaker with a sarcastic occupation are better understood as ironic and judged as more mocking than ironic statements uttered by a speaker with a non-sarcastic occupation (44). In other words, this means that the knowledge of social stereotypes, such as the speaker’s occupation, influences the extent to which we attribute ironic intent. In the present study, like HC participants, SZ patients seemed also sensitive to such social knowledge, showing different performances according to the speaker occupation. This result is in line with those of previous studies showing such sensitivity to contextual information (21, 30, 32, 36, 53). More specifically, Varga et al. (53) found that a linguistic help inserted into the context improves the SZ patients’ ability to understand irony. Indeed, SZ patients performed as well as HC participants when a word cueing irony (e.g., Sarah *furiously* remarks: “You did a pretty haircut” meaning “you did an ugly haircut”) was added in the context, while they performed worse than the HC participants when the stimuli did not contain such linguistic help. In the present research, SZ participants were also able to use social contextual information to attribute ironic intent to the speakers in the stories. Thus, social knowledge, such as speaker occupation stereotypes, seemed to help them in understanding speaker ironic intent, probably because stereotypes are social knowledge that is automatically activated. They do not require inferences. Sarcastic occupations are associated to specific characteristics (e.g., speaker’s tendencies to be humorous, critical, insincere, and to have lower levels of education), which are activated automatically without any inference when the participant thinks of the occupation. Such occupation stereotypes contribute to the ironic environment (44).

This is also probably the reason why our result disagrees with those of Corrigan and Penn and colleagues showing that SZ participants had specific impairment in recognizing and in using abstract social knowledge requiring inferences from the social situation (41). Contrary to the abstract social knowledge tested by these authors, stereotypes are social knowledge that does not require inference. In addition, following the results of Hirschfeld et al. (47), such social knowledge would be acquired and used outside any social engagement, in contrast to the abstract knowledge tested by Corrigan and Nelson (41). For these reasons (automaticity and apart from social engagement), SZ participants would be able to attribute ironic intent depending on the speaker occupation stereotypes.

### The case of mockery rating

Surprisingly here, by contrast with irony rating, SZ patients rated the extent to which the speaker was mocking someone or not in the same way (around 4), the statement being ironic or literal and the speaker occupation being sarcastic or not. To our knowledge, no research has investigated how SZ patients understand or feel mockery. Our result is probably to be linked with research on emotion recognition and emotion judgment by SZ patients since irony is used to express negative emotion (55). There is a general consensus in the literature on SZ processing of emotion, reporting that SZ participants have more difficulties in recognizing and judging the negative emotions than the positive ones (56). In these studies, SZ participants had to attribute an emotional state to a face, for example, while in our study, participants had to attribute negative attitude and emotion to the speaker. The attribution of a negative attitude to the speaker is probably more explicit in the case of mockery rating than in the case of irony rating, as it is the case when SZ participants are asked to judge emotions of a face. This hypothesis would account for the bad performance of SZ participants when they had to judge mockery while they succeeded in judging irony.

In addition, research has also shown that SZ patients with positive symptoms exhibited this specific impairment regarding negative emotion (57). However, our data do not support this specific profile since we did not find any correlation between mockery rating and the positive symptoms measured with the PANSS.

### Social perception using politeness rating

Social perception was assessed through the recognition of politeness. Participants were asking to rate the extent to which the speaker of the story was saying something polite or not. To our knowledge, none of the tests previously used to investigate social perception in SZ assessed politeness perception. The main results showed that SZ patients and HC participants rated politeness in the same way. Indeed, they rated the ironic statements as more polite than the literal ones. And statements uttered by a speaker with a non-sarcastic occupation were rated as more polite than statements uttered by a speaker with a sarcastic occupation. These results confirm those of Pexman and Olineck (44) and suggest that SZ patients did not have difficulties with social perception.

Although politeness is part of the rules that govern social situations referring to abstract social cues as defined by Corrigan et al. (40), it was correctly identified by the SZ patients in our study. Such finding diverges from the results of some of the studies by Corrigan and colleagues (39, 43). However, it is consistent with the result obtained by Corrigan et al. (40) showing that, in contrast to SZ inpatients, SZ outpatients are able to correctly identify both abstract and concrete cues. Indeed, our study only included outpatients. Such absence of failure may be due to symptom remission, as suggested by Corrigan et al. (40). Given that an effect of the speaker occupation was also found in both SZ and HC group, it is also possible that speaker occupation stereotypes help SZ patients to rate politeness.

In conclusion, the present study showed that individuals with SZ may be able to attribute mental states, such as speaker ironic intent, to a protagonist while they find it difficult to explicitly judge and attribute negative attitude, as evidenced by mockery rating. These findings provide a meaningful argument that the ability to attribute intention to others varies as a function of contextual information processing in SZ. While SZ patients fail to integrate non-social contextual information to attribute ironic intent (21), this attribution may be facilitated by social contextual information, such as stereotypes, whose distinctive feature is that they are automatically activated. We have to notice that social context information and particularly stereotypes on the speaker’s occupation concerns “the others” rather than “the other” as an individual. Thus, during an in-live communication, if a SZ patient knows that that the interlocutor has this or that occupation it may cue the attribution of ironic intent to his/her interlocutor. But other kind of contextual information, such as information on his/her interlocutor as a specific individual, will not be integrated leading to disturbance in the interaction.

Results of the present study have important consequences in terms of cognitive remediation since ToM ability could have an impact on social functioning in SZ (1, 3). The integration of contextual information seems to be a good target for cognitive remediation aiming to increase the social cognition ability.

## Author Contributions

All authors significantly contributed to the conception of the study, the analysis, and the interpretation of the data. All authors contributed to the final version of the manuscript.

## Conflict of Interest Statement

The authors declare that the research was conducted in the absence of any commercial or financial relationships that could be construed as a potential conflict of interest.

## Supplementary Material

The Supplementary Material for this article can be found online at http://journal.frontiersin.org/article/10.3389/fpsyt.2015.00098
