# Supplementary material for: Theory of Mind and Context Processing in Schizophrenia: The Role of Social Knowledge
Source: Front Psychiatry. 2015 Jul 3;6:98. doi: 10.3389/fpsyt.2015.00098 (PMC4490214; doi:10.3389/fpsyt.2015.00098)
Supplement: Supplementary file 1 [file Data_Sheet_1.PDF]

Appendix

Sample stimuli

<u>Story with non-sarcastic occupation (N= 8 x 2)</u>

A *veterinarian* (**non-sarcastic occupation**) sees Joannie arriving at work on Monday morning. Joannie seems to be a little bit more tired than usual. At midday, the *veterinarian* says to Pierre:

Joannie Looks well (ironic statement)/

Joannie does not look well (literal statement)

<u>Story with sarcastic occupation (N= 8 x 2)</u>

Marie said to her friend, *an actor* (**sarcastic occupation**), that she could memorize a poem of 20 lines in 5 minutes. Marie recited only half of the poem, forgetting the rest. The next day, the *actor* said to Guillaume:

Marie has a phenomenal memory (ironic statement)/

Marie has a poor memory (literal statement)

<u>Fillers (N= 8 x 2)</u>

Louise (**no occupation**) is moving house today and Michel told her that he will come to help her. Michel comes to help Louise but just for a few minutes. The following day, *Louise* says to Amélie:

Michel is helpful (ironic statement)/

Michel is uncooperative (literal statement)
